# Evaluation of Carbamazepine (CBZ) Supersaturatable Self-Microemulsifying (S-SMEDDS) Formulation *In-vitro* and *In-vivo*

**Published:** 2012

**Authors:** Zhang Nan, Gao Lijun, Wang Tao, Quan Dongqin

**Affiliations:** Institute of Pharmacology and Toxicology, Academy of Military Medical Sciences, Beijing,100850, China.

**Keywords:** S-SMEDDS, Carbamazepine, Bioavailability, Supersaturated state

## Abstract

The supersaturatable self-microemulsifying drug delivery system (S-SMEDDS) represents a new thermodynamically stable formulation approach wherein it is designed to contain a reduced amount of surfactant and a water-soluble polymer (precipitation inhibitor or supersaturated promoter) to prevent precipitation of the drug by generating and maintaining a supersaturated state *in-vivo*. The supersaturatable self-microemulsifying drug delivery system (S-SMEDDS) of CBZ was evaluated *in-vitro *and *in-vivo*. Three different formulations of CBZ were prepared and drug precipitation behavior, dissolution rate *in-vitro *and particle size distribution were evaluated. Studies on CaCO-2 permeability of three formulations were also carried out. Pharmacokinetic studies were conducted in beagle dogs with administration dose of 200mg to assess bioavailability *in-vivo *compared with commercial tablet. The results showed that the presence of a small amount of polymeric precipitation inhibitor (PVP) effectively sustained supersaturated state by retarding precipitation kinetics. The mean particle size after dispersion was about 33.7 nm and the release rate from S-SMEDDS was significantly higher than the commercial tablet *in-vitro*. S-SMEDDS formulation with precipitation inhibitor decreased impairment to cells due to a lower surfactant level compared to SMEDDS. The absorption of S-SMEDDS *in-vivo *resulted in about 5-fold increase in bioavailability compared with the commercial tablet and the reproducibility of plasma concentration profiles intra-individual was improved remarkably. This study demonstrates that S-SMEDDS technology provide an effective approach for improving the extent of absorption of poorly-soluble drugs with low level of surfactant.

## Introduction

Carbamazepine (CBZ) is an antiepileptic drug of “Class II” in the Biopharmaceutical Classification System with polymorphs and low solubility in water (113 μg.m/L, 25°C) (1) exhibiting slow and irregular gastrointestinal absorption (2). Considerable variability in CBZ plasma concentration was also reported (3-5). Several attempts have been made to enhance the bioavailability of CBZ utilizing a solid dispersion system with sugar or polyethylene glycols (PEG6000), co-precipitation with phospholipids and complexation with hydroxypropyl-cylcodextrin (HP-*β*-CD) (6-8). CBZ tablets stored in humid conditions often did not offer adequate seizure control due to the formation of CBZ dihydrate (9, 10).

Self-microemulsifying drug delivery system (SMEDDS) can be defined as an isotropic multi-component drug delivery system composed of surfactant, co-surfactant and oil, which spontaneously form microemulsion in the presence of water (11-12). Conventional self-microemulsifying drug delivery systems (SMEDDS) are widely used in enhancing the oral absorption of poorly-soluble drugs (13-16). When SMEDDS formulations introduced into gastrointestinal area (GI), drug precipitation may be occurred and lead to failure of improvement of intestinal absorption. On the other hand, high surfactant level typically present in SMEDDS formulations can cause GI side-effects. The supersaturatable self-microemulsifying drug delivery system (S-SMEDDS) represents a new thermodynamically stable formulation approach wherein it is designed to contain a reduced amount of surfactant and a water-soluble polymer (precipitation inhibitor or supersaturated promoter) to prevent precipitation of the drug by generating and maintaining a supersaturated state *in-vivo*. The S-SMEDDS formulations can result in enhanced oral absorption as compared with the related self-emulsifying drug delivery systems (SMEDDS) formulation and the reduced surfactant levels may minimize gastrointestinal surfactant side effects (17-20).

In S-SMEDDS, solubilization of CBZ in o/w microemulsions will enhance its bioavailability with low surfactant level and CBZ can be absorbed in GI homogeneously, which may reduce variability in plasma concentration. The main objectives of the study were to develop S-SMEDDS of CBZ to improve its dissolution *in-vitro *and bioavailability as well as reducing the intra- and inter subject variability of the blood level of CBZ *in-vivo*.

## Experimental

*Materials and apparatus*

API of CBZ was purchased from Jiuzhou pharmaceutical Industrial Co.(China), Tween 80 and PEG400 were obtained from sigma Chem. Co. and medium chain triglycerides (MIGLYOL 812N) were supplied by Gatte-fosse (France); Cremphor EL-35 was from BASF (Germany), and PVPK30 was from ISP (USA). S-SMEDDS of CBZ were prepared by our lab and CBZ tablets were purchased from Shanghai Sanlian Pharma Ltd. (batch No.20061101). Beagle dogs were from animal center of Academy of Military Medical Sciences 13-15 Kg male.

Hermle Z200A high speed centrifugator was from Germany, Hitachi L-7100, HPLC was from Japan, QL-901 vortex mixer came from Shanghai, China UV-160A vis-ultraviolet spectrophotometer was from SHIMAZU Co. Tokyo, Japan, and ELECTROLAB TDT-08L drug dissolution test instrument was made in ELECTROLAB corporation, U.S.A.

*Different formulations of CBZ*

Three formulations with or without precipitation inhibitor were prepared as showed in Table 1. 

**Table 1:** Composition of S-SMEDDS formulations

**S-SMEDDS**	**Composition (%, w/w)**			
	**oil**	**cosurfactant**	**surfactant**	**precipitation inhibitor**
Formulation A	37	15	45	0
Formulation B	40	20	35	2
Formulation C	40	22	35	0

*Each formulation contained 3% CBZ

Formulation A (SMEDDS) with high level of surfactant without precipitation inhibitor, Formulation B (S-SMEDDS) with low level of surfactant with 2% (w/w) precipitation inhibitor and Formulation C (SMEDDS) with low level of surfactant without precipitation inhibitor were compared and evaluated.

*In-vitro evaluation of precipitation*

The performance of three formulations of CBZ was evaluated with respect to drug precipitation when contacted with an aqueous medium. 0.1 mol/L HCl was chosen as the medium *in-vitro. *One gram of the S-SMEDDS formulations was placed into 50 mL of the medium and stirred at 50 r/min. About 0.5 mL solution samples were taken at 0, 4, 8, 12, 24 and 36 h and the filtrate were analyzed by HPLC at wavelength of 285 nm.

*Dissolution in-vitro*

A CP2005 apparatus with baskets rotating at 50 r/min was used. A volume of 0.1 mol/LHCl (900 mL) was used as the dissolution medium and maintained at 37 ± 0.5°C.The market tablets and S-SMEDDS formulation of CBZ ( formulation B) in soft capsule were positioned in the basket at t = 0 min. Samples of 5 mL were withdrawn at various intervals of time (5, 10, 15, 20, 30, 45, min for S-SMEDDS formulation, at 10, 20, 30, 45, 60 min for tablets) and the samples was filtered by 0.22 μm filter film. The volume of the dissolution medium was kept constant throughout the run by replacing the removed samples with an equal quantity of freshly solution. In all cases, three runs were carried out for each formulation. The accumulated amount of drug released at each sampling point was corrected with the volume of the dissolution medium. The concentration of CBZ in each samples were analyzed spectrophotometrically at wavelength of 285 nm.

*Emulsion droplet size analysis*

Formulation B-2 (1 g) was diluted with purified water at 37°C with a stirring rate of 50 r/m using a rotating paddle dissolution apparatus. The droplet size distribution of the resultant emulsions after 20 min was determined by a laser diffraction sizer (Zetasizer 3000HS, UK), with the ability of measuring sizes between 10 and 5000 nm.

*CaCO-2 cell permeability study*

*Cells culture and recovery*

CaCO-2 cells, originating from a human colorectal carcinoma, were provided by ATCC (American Type Culture Collection). For these transport studies, cells at passage 50 were used and allowed to grow and differentiate to confluent monolayer for 21 days. Confluence and differentiation of the cell monolayer on inserts was measured through evaluation of the Trans-Epithelial-Electrical-Resistance (TEER). (EVOM, World Precision Instruments, Sarasota, FL). Inserts containing PBS buffer ( pH = 7.2) alone served as a negative control. At the end of the experiment, PBS was replaced by growth medium. Inserts were incubated for an additional 48 h for recovery TEER measurements. Inserts with TEER-values > 600Ω•cm^2^ were considered as recovered. The average TEER-values were 654 ± 43Ω•cm^2^.

*Permeability studies*

Prior to the experiment, the culture medium was removed, cells were washed with PBS and incubated for 30 min at 37°C, 45 r/m. The filter-grown monolayers of 21–23 days of age were exposed to different CBZ S-SMEDDS (Table 1) within a period of 2 h. Transport studies were performed at 37°C, with shaking at 45 r/m (n = 3). Subsequently, samples (100 μL) were taken from the receiver side at specified time intervals and replaced with an equal volume of PBS. The aliquots collected at various intervals of time were analyzed by HPLC.

*Pharmacokinetic behavior in beagle dogs*

The study was approved by the Ethical Committee of academy of military medical sciences. Six beagle dogs, weighting 13-15 Kg, male, were obtained from animal center of academy of military medical sciences (Beijing, China). All animals were housed individually in standard cages on a 12 h light-dark cycles and were fed with standard animal chow daily and had free access to drinking water

*Experimental protocol*

After an overnight (12 h) fasting, six beagle dogs were administered orally with the dose of 200 mg per dog. According to Table 2, A is commercial tablet, and B is S-SMEDDS formulation(Formulation B). Dogs were allowed free access to water after dosing for 4 h. Approximately 3 mL blood samples were collected into heparinized tube at 0.25, 0.5, 0.75, 1.0,1.25,1.5,2.0,4.0, 6.0, 8.0,10.0,12.0 h, Plasma was separated by centrifugation at 3500 r/min for 20 min and kept frozen at -20°C until analysis. The concentration of CBZ in plasma was determined by HPLC. The washout period was one week.

**Table 2:** Drug administration to beagle dogs

**Administration period **	**Animal number**
	1 2 3 4 5 6
1	A B A B A B
2	B A B A B A

*Determination of CBZ concentration in plasma*

The mobile phase consisted of methanol-water (57:43, v/v), at a flow rate of 1 mL/min. The column used was ZORBAX SB-C18 (4.6 mm×150 mm, 5 μm).The detection wavelength was 285 nm. 50 μL of diazepam(10.0 μg/mL) internal standard solution was added to 0.5 mL plasma in a 10 mL glass centrifuge tube for assay and then 3 mL ethylether was added, the samples were shaken for 5 min by vortex mixer and centrifuged at 3000 r/min for 10 min. The supernatant phase was separated and evaporated to dryness at 40°C under nitrogen. Residues were dissolved in 100 μL mobile phase and were assayed by HPLC.

*Data analysis*

Pharmacokinetic analysis was performed by means of a model independent method using DAS2.0 computer program (issued by the State Food and Drug Administration of China for pharmacokinetic study). The area under the plasma concentration versus time curve from zero to 12 h (AUC_0~12h_) was calculated by linear trapezoidal rule from zero to the last plasma concentration. The maximum plasma concentration, *C*_max_, and the time of its occurrence, *T*_max_, were compiled from the concentration–time data. All results were expressed as mean ± SD. The data from different formulations were compared for statistical significance by one way analysis of variance (ANOVA). T-tests were performed to evaluate the significant differences between the two formulations and the data were considered statistically significant at p < 0.05.

## Results and Discussion

*In-vitro evaluation of precipitation*

Based on the results of Table 3, the drug precipitation can be prevented and CBZ kept solubilized for a prolonged period of time(over 24 h) when PVPK90 was added as precipitate inhibitor in the formulation at low surfactant level, while drug precipitation occurred within 4 h when formulating without precipitate inhibitor. S-SMEDDS may generate a protracted supersaturated solution of the drug with a reduced surfactant level when the formulation is released into an aqueous medium. 

**Table 3:** The content(w/w, %) of CBZ S-SMEDDS after dilution

**Formulations **		**Time (h)**						
		0	4	8	12	24	36	72
Formulation A	100		99.9	99.7	80.5	68.3	—	—
Formulation B	100		99.9	99.7	100.3	99.8	75.5	54.3
Formulation C	100		70.2	48.7	—	—	—	—

*Dissolution test in-vitro*

**Figure 1:**
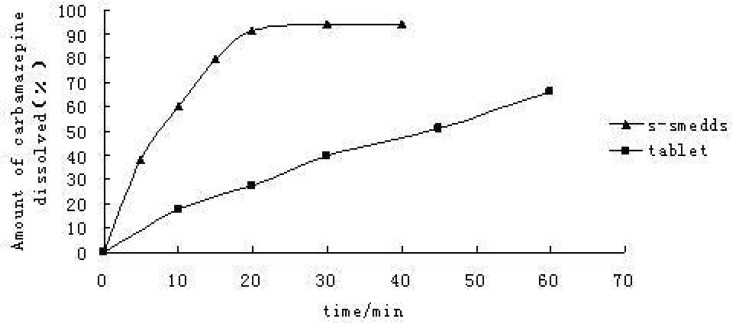
Dissolution curve of CBZ preparations *in-vitro *(n = 6).

Dissolution curve Figure 1 showed that over 90% of CBZ dissolved from the self-emulsifying formulations within 20 min, behaving like a fast-dissolving immediate release drug product, and from the commercial tablets, only 30% of the dose was released within 20 min. The self-emulsifying systems can significantly increase CBZ dissolution rate *in-vitro.*

*Caco-2 cell permeability study*

**Figure 2:**
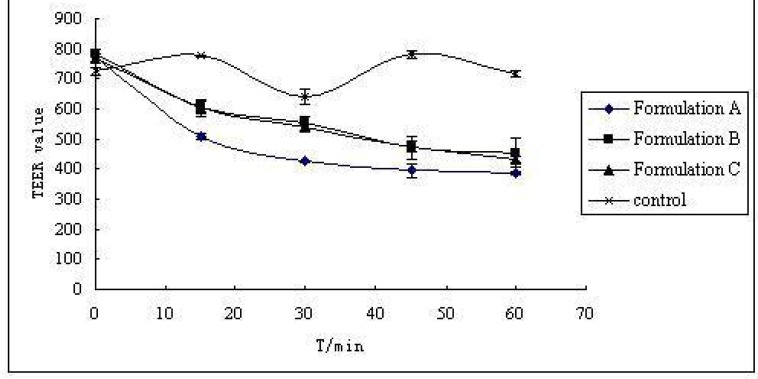
TEER-value of CaCO-2 cell with different formulations (n = 3

**Figure 3:**
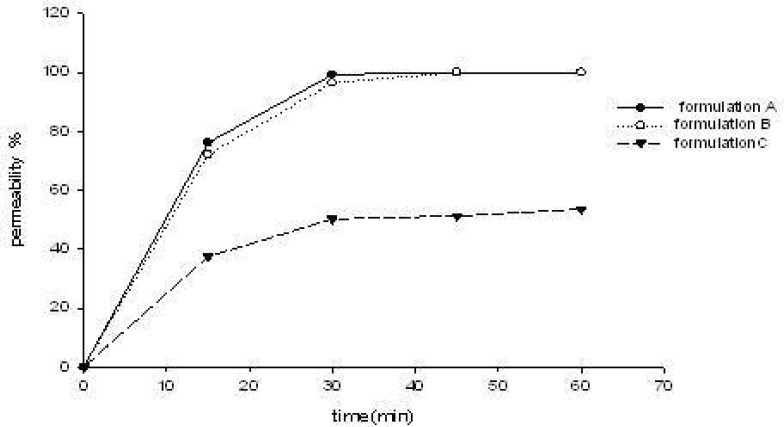
Permeability of CBZ across CaCO-2 cells with different formulations (n = 3).

The three S-SMEDDS formulations were presumed to enhance CBZ transport across the cell membrane. Increased drug absorption through the intestinal mucosa is often associated with damage caused to the intestinal cells and to their barrier function. The effect of different CBZ formulations on the monolayer integrity was examined by measuring the TEER-value before and after treatment of CaCO-2 cell monolayers with three formulations (formulation A, B, C of Table 1) for predetermined time intervals of 15, 30, 45 and 60 min. As demonstrated in Figure 2, the results showed a reduction in TEER-values after the experiment. This implied that S-SMEDDS may affect the paracellular route through the opening of tight junctions and thus reduce the cell integrity of Caco-2 cells. However, TEER-values measured 48 h after transport experiment (recovery) revealed that all the monolayers fully recovered (TEER-values were higher than 600 Ω•cm^2^). This indicated that although the formulations affected the tightness of the cell monolayer, it reversibly recovered after the experiment. On the other hand, the TEER-value of monolayers reduced remarkably when treated with formulation A (high surfactant concentration, p < 0.05), and there was no significant differences between formulation B and formulation C (p > 0.05). From the results of permeability of CBZ across CaCO-2 cell mono-layers for 15, 30, 45 and 60 min( Figure 3), it can be observed that they were not statistically different when treated with formulation A and formulation B (p > 0.05), but had significant differences between formulation B and formulation C (p < 0.05). With the same drug permeability, formulation B (with precipitation inhibitor (S-SMEDDS)) decreased impairment to cells due to a lower surfactant level compared to formulation A (SMEDDS) with high surfactant concentration.

*Pharmacokinetics in beagle dogs*

**Figure 4:**
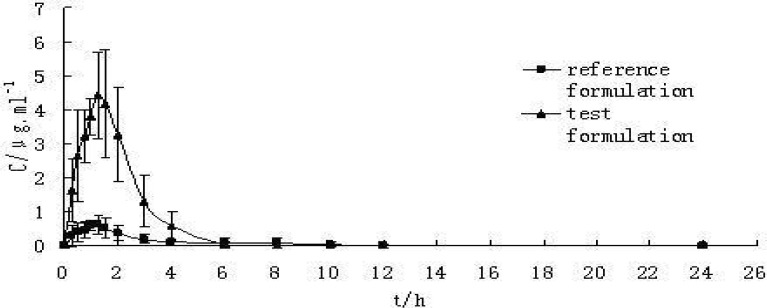
Plasma concentration-time curve of CBZ preparations in beagle dogs(n = 6) Reference formulation with commercial tablet (200 mg) and test formulation with S-SMEDDS (200 mg

The maximum plasma concentration (C_max_) and the corresponding peak time (t_max_) were determined through the inspection of the individual drug plasma concentration-time profiles. AUC_0-t_ was calculated by the linear trapezoidal rule, and AUC_0-∞_was calculated as AUC_0-t_+C_t_ / K_e_. The results are showed in Table 4. The statistic analysis of pharmacokinetic data through Paired t-test showed that AUC_0-t_, and C_max_ had significant differences between S-SMEDDS formulation and the commercial tablet. Plasma concentration-time curve of CBZ commercial tablet (reference) and S-SMEDDS (test) in beagle dogs (n = 6) can be seen in Figure 4. The absorption of S-SMEDDS *in-vivo *resulted in about 5-fold increase in bioavailability compared with the conventional tablet and the reproducibility (RSD%) of plasma concentration profiles intra-individual was improved remarkably. The higher absorption characteristics of CBZ from the S-SEDDS formulation are attributed to a high free drug concentration *in-vivo*, implying a supersaturated state. So it was concluded that S-SMEDDS of CBZ enhanced its bioavailability with low surfactant level and CBZ can be absorbed in GI homogeneously which may reduce variability in plasma concentration. 

**Table 4:** Pharmacokinetic parameters of CBZ preparations in beagle dogs (n = 6).

**Parameter**	**Tablets**	**S-SMEDDS**	**P**
C_max_ (mg/L)	0.74 ± 0.19	4.96 ± 1.16**	< 0.001
AUC_0-t_(mg.h/L)	1.67 ± 1.19	9.83 ± 2.47**	< 0.001
t1/2z (h)	1.28 ± 0.49	0.73 ± 0.31	> 0.05
MRT0-∞ (h)	2.14 ± 1.33	1.69 ± 0.40	> 0.05

**:Compare with tablets by paired t-test

*In-vitro evaluation of precipitation and dissolution*

An *in-vitro *precipitation screening study was used to direct development of a novel S-SMEDDS formulation of CBZ. Determination of the true free drug concentration of CBZ *in-vitro *test medium was analytically challenging. The drug may exist in any one of four different states, i.e., free molecules in solution (free drug completely monomolecularly dissolved), supersaturated solubilized molecules or “compound” with precipitation inhibitor (interacted with excipients), molecules partitioned into microemulsions and precipitated solid particles. It is difficult to separate the drug in each phase; in this case, dissolved CBZ which was determined in the dissolution and precipitation test contains undissolved small droplets and particles through the 0.22 μm filter. On the other hand, the particle size of the precipitated solids grew significantly during the course; the actual degree of supersaturation at any given time was difficult to determine precisely.

*In-vivo bioavailability*

SMEDDS and S-SMEDDS are systems which can form fine oil−in−water (o/w) microemulsions in the gastrointestinal tract. This formation of fine droplets provides a large surface area for pancreatic lipase to hydrolyse triglycerides and thereby promots a rapid release of the drug substance and/or the formation of mixed micelles containing the drug substance. Greater availability of dissolved CBZ from the SMEDDS or S-SMEDDS formulation could lead to higher absorption and higher oral bioavailability. However, we have not performed *in-vivo *study for the comparison with conventional SMEDDS. From the evaluation results of precipitation *in-vitro*, SMEDDS formulation without precipitation inhibitor at low surfactant level was not stable with occurrence of drug precipitation within 4 h. S-SMEDDS with the presence of a small amount of polymeric precipitation inhibitor (PVP) effectively sustained supersaturated state by retarding precipitation kinetics. When SMEDDS formulations are introduced into gastrointestinal area(GI), drug precipitation may occur and lead to failure of improvement of intestinal absorption. On the other hand, high surfactant level typically present in SMEDDS formulations can cause GI side-effects. The supersaturatable self-microemulsifying drug delivery system (S-SMEDDS) represents a new thermodynamically stable formulation approach wherein it is designed to contain a reduced amount of surfactant and a water-soluble polymer (precipitation inhibitor or supersaturated promoter) to prevent precipitation of the drug by generating and maintaining a supersaturated state *in-vivo*. In S-SMEDDS, solubilization of CBZ in o/w microemulsions will enhance its bioavailability with low surfactant level and CBZ can be absorbed in GI homogeneously which may reduce variability in plasma concentration.

## Conclusion

S-SMEDDS of CBZ were developed and their drug precipitation behavior, CaCO-2 cell permeability, dissolution rate *in-vitro *and bioavailability in beagle dogs *in-vivo *were evaluated. The results demonstrate that S-SMEDDS technology provide an effective approach for improving the extent of absorption of poorly-soluble drugs with low surfactant level and decreased impairment to cells compared to SMEDDS.
